# Cadmium exposures and deteriorations of cognitive abilities: estimation of a reference dose for mixture risk assessments based on a systematic review and confidence rating

**DOI:** 10.1186/s12940-022-00881-9

**Published:** 2022-07-14

**Authors:** Mousumi Chatterjee, Andreas Kortenkamp

**Affiliations:** College of Health, Medicine and Life Sciences, Brunel University London, Centre for Pollution Research and Policy Kingston Lane, Uxbridge, UB8 3PH UK

**Keywords:** Cadmium toxicity, Systematic review, Developmental neurotoxicity, IQ, Reference dose, Mixture risk assessment

## Abstract

To support a mixture risk assessment with a focus on developmental neurotoxicity we evaluated the strength of evidence for associations of cadmium exposures with declines in IQ by conducting a systematic review and confidence rating. We searched peer-reviewed studies published in English between 2012 and July 2021 and identified 15 eligible studies (11 prospective cohort studies, and 4 cross-sectional studies). Of the 10 studies that observed associations of cadmium exposure with child IQ declines, two achieved an overall “High (H)” confidence rating, five a “Medium to High (M/H)”, one a “Medium (M)” and two a “Low (L)” confidence rating. Five studies did not detect significant associations between cadmium exposure and reduced cognitive ability; of these, two received a “High (H)” confidence rating, two an overall rating of “Medium to High (M/H)” and one a “Medium (M)” rating. The null findings reported by the “High (H)” and Medium to High (M/H)” studies could partly be explained by low exposures to cadmium or confounding with high levels of lead. By using a one-compartment toxicokinetic model in a reverse dosimetry approach, we estimated that a daily intake of 0.2 μg/kg body weight/day corresponds to urinary cadmium levels no longer associated with cognitive declines observed in a “High (H)”-confidence study. This estimate is 1.8-fold lower than the current health-based guidance value (HBGV) for kidney toxicity of 0.36 μg/kg bodyweight/day established by the European Food Safety Authority (EFSA). Our value does not have the normative character associated with health-based guidance values and is intended only as a reasonable estimate for the purpose of mixture risk assessments. However, with cadmium exposures in Europe between 0.28 (middle bound) and up to 0.52 μg/kg bodyweight/day (95^th^ percentile), our review suggests that pregnant women and children are poorly protected against neurodevelopmental effects. This warrants a revision of the current HBGV.

## Background

Heavy metals and metalloids including lead, methylmercury, arsenic, and manganese are well-known neurotoxicants [34], along with other chemicals such as organophosphate pesticides [15, 27], polybrominated diphenyl ethers [4, 12] or polychlorinated biphenyls [20]. These substances are ubiquitous in the environment and occur widely in food, water, air, and consumer products. Systematic investigations of simultaneous exposures to these chemicals and their effects on brain development are therefore necessary, even more so since the economic costs of these effects can be very high. Based on relatively few chemicals (polybrominated diphenyl ethers, organophosphate pesticides) a 2015 study has estimated that the costs associated with declining cognition (loss of IQ points) due to exposure to neurotoxic chemicals amount to 155.44 billion euros annually in the European Union alone [2].

This paper addresses whether cadmium should be included in a mixture risk assessment of developmental neurotoxicants, with a focus on declines in cognitive abilities, measured as IQ. Cadmium exposure is widespread; among non-occupationally exposed populations and non-smokers, the diet is the main exposure source. Cereals and root vegetables contribute most of the cadmium intake via food, and high consumption of seafood and smoking can add significantly to the load ([7] and [8]).

As the placenta cannot completely block the transfer of cadmium from the mother to the fetus [28], exposure to cadmium starts early in life. It is therefore conceivable that cadmium can disrupt brain development, a possibility that suggested itself based on experimental studies in animals [39]. However, in a systematic review of human studies exploring associations between cadmium exposures and neurodevelopmental effects Rodriguez-Barranco et al. [32] found that most available studies were inconclusive, except for Tian et al. [38]. In this Chinese cohort, significant declines in full-scale intelligence quotients (FSIQ) among 4.5-year-old children were associated with maternal cadmium exposures measured as concentrations in pregnancy- and cord blood. Since then, further studies appeared that demonstrated associations of prenatal cadmium exposures with poor cognition [17, 18], but others [10] were unable to observe these links. There are also indications that postnatal cadmium exposures early in a child’s life might have detrimental effects on cognition [33]. In a more recent systematic review of studies of prenatal cadmium exposures and cognitive development, Liu et al. [24] concluded that there is convincing evidence of harmful effects on the cognitive abilities of offspring. However, the review by Liu did not consider the impact of cadmium exposures in early childhood. These exposures are usually higher than those experienced prenatally and could therefore also influence childrens’ cognitive development. Furthermore, several large studies appeared after 2018, the census date of Liu’s review.

In this paper we evaluate whether it is possible to estimate a reference dose for cadmium exposures and deteriorations of cognitive abilities for the purpose of a mixture risk assessment, and if so, attempt such a derivation. To provide a sound basis for this effort, we conducted a review that includes cadmium exposures in early childhood and considered studies that appeared up until 2021, building on the work by Rodríguez-Barranco et al. [32] and Liu et al. [24].

A widely used approach for the assessment of combined exposures to hazardous chemicals is the Hazard Index (HI) method [37]. For all chemicals included in the assessment, it builds risk quotients of exposure and health-based guidance values, or reference doses. By adding up these risk quotients, it examines exceedances of "acceptable" combined exposures relative to an index value of '1'. To achieve consistency in this assessment, the risk quotients must be built with reference doses for similar, if not identical toxicities. A mixing of reference doses for different toxicities should be avoided as this will introduce uncertainties in the assessment.

Addressing the issue of a reference dose for cadmium neurodevelopmental toxicity therefore requires a decision about the specific endpoint that should be chosen for the assessment. Existing health-based guidance values for other developmental neurotoxicants such as lead, methylmercury, and PCBs are all based on IQ declines. We therefore attempted to estimate a cadmium reference dose for IQ declines because of developmental and early childhood exposures.

Cadmium is primarily known for its adverse effects on the kidney (especially to proximal tubular cells), causing renal dysfunction. In 2009 a revised health-based guidance value of 0.36 μg/kg/day for cadmium was established by the CONTAM Panel of the European Food Safety Authority [7]. Investigations also demonstrated the endocrine disrupting [6] and estrogenic activities of cadmium [30, 35]. Cadmium is also known for its endocrine disrupting activities and thyroid toxicity due to its binding affinity with cysteine-rich proteins, metallothioneins, in the thyroid gland (Klassen et al. [19]).More recently, cardiovascular and bone toxicity have received critical attention [22]. However, neither the existing guidance value [7] nor the new estimate of 0.35 μg/kg/day for bone toxicity [22] can be relied on for a mixture risk assessment based on IQ loss in children.

## Materials and Methods

Literature search and screening, study evaluation, data extraction and evidence synthesis methods are set out in detail in the systematic review protocol that we developed following the COSTER recommendations (10.1016/j.envint.2020.105926). Briefly, epidemiological studies with cadmium that investigated losses in IQ scores were identified by conducting literature searches in PubMed, Web of Science, Scopus until July 2021. Citation searches for key papers were also conducted.

### Search Strategy and inclusion criteria

The PECO (Population, Exposure, Comparator, Outcome) statement and details of our search strategy are available through the study protocol (10.5281/zenodo.5767669). We used the search syntax: ("cadmium" OR "Cd") AND ("cognitive ability*" OR "intelligence" OR "IQ" OR "cognitive skills" OR "mental abilit*" OR "cognitive function*" OR "cognitive performance" OR "intelligence quotient" OR "general mental ability" OR "cognitive capacity" OR "mental capacit*" OR "intellectual function*").

We included epidemiological studies of pregnant women and their children who were exposed to cadmium through the diet published after 2012 and up until July 2021. We first examined the strength of evidence for associations between cadmium and IQ declines and assessed studies that measured cadmium in urine and blood. Urinary cadmium levels reflect the total body burden accumulated during a lifetime. Cadmium levels in pregnancy blood or cord blood are indicative of exposures during the previous 3 months and can be a reliable marker of prenatal and perinatal exposures [22]. We excluded studies that measured cadmium concentrations in hair as the exposure marker, because these depend on hair length, and therefore only reflect relatively recent exposures in people with short hair. There are also complications due to the possibility of external contamination, and sufficient information about typical concentration ranges in the general population is missing (see the discussion in [33]). For estimations of a reference dose protective of exposures associated with IQ declines, we considered studies that measured cadmium concentrations in urine.

We focused on studies that used relevant editions and variations of the Wechsler Preschool and Primary Scales of Intelligence (WPPSI), the Bayley Scale of Development (BSD), the McCarthy Scales of Childrens’ Abilites (MSCA) or the Gesell Development Domains. We excluded human studies that involved other developmental neurotoxicity endpoints such as behavioural studies, autism, ADHD (attention deficit hyperactivity disorder), or motor activities. The exclusion and inclusion criteria are shown in the review protocol (10.5281/zenodo.5767669). The literature review process was coordinated and managed using the freely available CADIMA tool (https://www.cadima.info/index.php/area/evidenceSynthesisDatabase). Title, abstract, full-text screening, and data extraction was performed by two reviewers.

### Study Evaluation

We assessed the Risk of Bias (RoB) for human epidemiological studies by using predefined criteria and considerations. The two main concerns were the RoB (understood as factors that affect the magnitude or direction of effects and compromise the internal validity of a study) and insensitivity (factors that limit the ability of a study to detect an effect that is, in fact, present).

We examined eligible studies of associations between cadmium and IQ declines using the procedures detailed by Radke et al. [31], with the following evaluation domains: exposure measurement, outcome measurement, participant selection, confounding and analysis. By applying the detailed criteria of Radke et al. [31], we judged each evaluation domain regarding its utility for hazard identification with the categories “Good (G)”, “Adequate (A)”, “Poor (P)” and “Critically Deficient (CD)”. The rating of “Good (G)” was applied when the evaluation domain demonstrated appropriate study conduct with only minor deficiencies not expected to influence the study results. “Adequate (A)” was assigned wherever there were some limitations, but when these were unlikely to have an impact on study outcomes. We used “Deficient (D)” when shortcomings were judged to have a substantial impact on results or to prevent reliable interpretation of studies. “Critically deficient” was chosen for evaluation domains with serious flaws that made a study uninterpretable.

Accordingly, the exposure evaluation domain was judged as “Good (G)” when appropriately sensitive methods for the analytical determination of cadmium in blood or urine were used (usually atomic absorption spectroscopy, AAS, with graphite furnace or inductively coupled plasma mass spectrometry, ICP-MS), with limits of detection (LOD) reported and evidence of quality assurance (QA) measures. “Adequate (A)” was used when information on LODs was missing, but when suitably sensitive analytical methods were used. Studies that did not meet these criteria were evaluated as “Deficient (D)” or below.

In the outcome domain, studies that employed recognised methods for measuring cognitive abilities (WPPSI, BSD, MSCA, Gesell or national adaptations), performed in conducive environments by qualified personnel were rated as “Good (G)”. When test outcomes were self-reported or not raised by qualified personnel, we applied the rating of “Deficient (D)”.

The participant selection aspect was assigned a rating of “Good (G)” when study subjects were drawn from the general population with no evidence of selection bias or loss to follow-up. When information on follow-up was missing, but without evidence of significant loss, we applied “Adequate (A)”. Where study participants were recruited from hospital populations we also chose “Adequate (A)”. Studies with loss to follow-up of more than 50% were rated as “Deficient (D)”.

The confounding domain was evaluated as “Good (G)” when all key confounders were considered (maternal IQ, maternal age, socio-economic status, home environment, smoking during pregnancy, pre-pregnancy body mass index (BMI), maternal diabetes, maternal education, breast-feeding, concentrations of lead and mercury in blood or urine). We applied “Adequate (A)” when some, but no key confounders were missing. Studies that did not consider confounding by maternal IQ or lead and mercury exposures were rated as “Deficient (D)”.

In the analysis domain, studies that evaluated cognitive performance on a continuous scale, with confounding accounted for were rated as “Good (G)”. Dichotomisations received an evaluation of “Adequate (A)”, and other shortcomings were rated as “Deficient (D)” or below.

We combined the assessments for each single evaluation domain to arrive at overall study confidence ratings of “High (H)”, “Medium (M)”, “Low (L)” or “Uninformative (U)”. We applied the following decision rules: Studies with all evaluation domains evaluated as “Good (G)” or no more than one assessed as “Adequate (A)” received an overall confidence rating of “High (H)”. When the participant selection domain received a rating of “Deficient (D)” due to loss of follow-up, but all other domains were assigned “Good (G)”, the overall confidence rating was “Medium to High (M/H)”. Similarly, we pegged the overall rating at “Medium to High (M/H)” when maternal IQ was not recorded (confounding domain) but where this was mitigated by the availability of other indicators of home environment or socioeconomic status, and when all other aspects were evaluated as “Good (G)”. An overall rating of “Medium (M)” was assigned when one evaluation domain received a “Deficient (D)” and another one “Adequate (A)”, with all other domains rated as “Good (G)”. With two domains classed as “Deficient (D)”, the overall confidence rating was “Low (L)”. We pegged the rating of studies that did not record lead, mercury or arsenic exposures or did not adjust for these exposures at “Low (L)”.

### Data synthesis

Due to data heterogeneity, a quantitative meta-analysis was not conducted. We therefore provided a narrative synthesis to summarise and explain the characteristics and findings of the included studies in terms of cadmium exposure ranges and IQ loss.

### Evidence synthesis

We first assessed whether the evidence of association of IQ loss and cadmium exposure from human epidemiological studies is sufficiently strong to support hazard identification. To address this question, we employed methods for weighing evidence from human studies, following the principles described in EFSA guidance [9]. With the Bradford Hill criteria as a guiding principle, the evidence was synthesized by considering aspects of an association that may suggest causation: consistency, exposure-response relationship, the strength of association, temporal relationship, biological plausibility, and coherence.

We adopted the criteria developed by Radke et al. [31] for arriving at strength of evidence conclusions in terms of “Robust (R)”, “Moderate (M)”, “Slight (S)”, “Indeterminate (I)” or “Compelling Evidence of No Effect (CENE)”. “Robust (R)” describes a situation where evidence from several “High (H)”, “Medium to High (M/H)” or “Medium (M)” confidence independent studies shows that cadmium exposures are associated with IQ loss, with reasonable confidence that alternative explanations, including chance, bias, and confounding, can be ruled out across studies. The evaluations of “Moderate (M)” is used where a smaller number of studies (at least one “High (H)” or “Medium (M)” confidence study with supporting evidence) demonstrate associations between cadmium and IQ declines, with some heterogeneous results, and where the degree of confidence required for “Robust (R)” was not reached. “Slight (S)” is assigned when one or more studies report an association between cadmium exposure and IQ loss but where considerable uncertainty exists. The evidence is limited to a set of consistent low confidence studies or higher confidence studies with unexplained heterogeneity. “Indeterminate (I)” is used when there are no studies available in humans or when the evidence is highly inconsistent and of low confidence. “Compelling Evidence of No Effect (CENE)” requires several high confidence epidemiological studies returning null results.

### Methods for estimating a reference dose

To derive a cadmium-reference dose associated with IQ loss in children, we first identified epidemiological studies with an overall confidence rating of “High (H)”, Medium to High (M/H)” or “Medium (M)”. The dose metric of daily intake (μg/kg body weight/day) is required to build risk quotients that utilise widely available estimates of daily or weekly cadmium intakes. We gave preference to studies that employed maternal or child urinary cadmium levels as the exposure marker, not only because this reflects a lifetime body burden, but also because reliable toxicokinetic models for the estimation of corresponding daily intakes are available (see below). For the biomarker blood, toxicokinetic models to work out corresponding daily intakes are not available.

We identified so-called points of departure – urinary cadmium concentrations no longer observed to be associated with IQ losses (epidemiological “no-observed-adverse-effect levels”). By using the one-compartment toxicokinetic model developed by Amzal et al. [1] we converted these urinary cadmium concentrations into corresponding daily intakes, with the following formula:

$${Cd}_{urine}(age)=\frac{f_u\times {f}_k}{\log (2)}\times d\times {t}_{1/2}\frac{\left[1-\exp \left(-\frac{\log (2)\times age}{t_{1/2}}\right)\right]}{\left[1-\exp \left(-\frac{\log (2)}{t_{1/2}}\right)\right]}$$

where *Cd*_*urine*_ *(age)* is the urinary cadmium concentration normalised for creatinine (μg Cd/g creatinine) at a specific age of the individual; *d* is daily dietary Cd exposure (μg/kg/day); *t*_*1/2*_ is the half-life of Cd in the renal compartment and *f*_*u*_ a constant that relates cadmium in the kidney cortex to urinary concentrations. *f*_*k*_ is a composite for the degree of cadmium absorption in the gastrointestinal tract, the fraction of cadmium transported to the kidney, a coefficient translating whole kidney cadmium into concentrations in the cortex, and kidney weight as a fraction of body weight [1]. Following Amzal et al. [1] we used the following values: The half-life was set as 11.6 years, *f*_u_*f_*k*_ was fixed at 0.005, and log (2) in the formula was taken as the natural logarithm with a value of 0.6931.

## Results

**Fig. 1:**
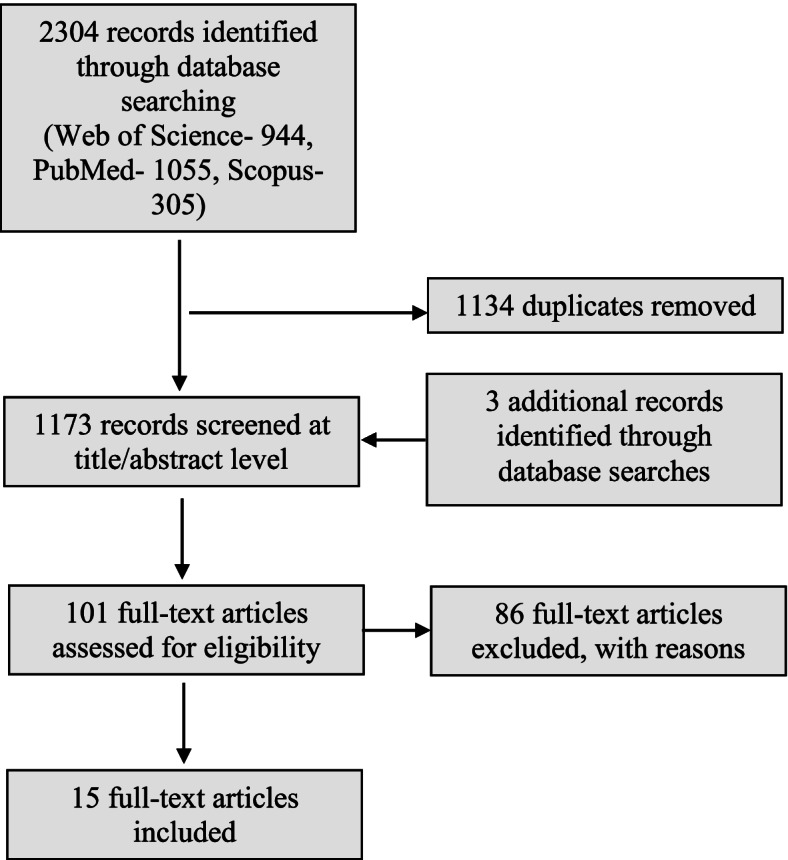
Selection of eligible full text records for systematic review of cadmium studies on declines in IQ

Our literature search yielded a total of 2304 records from PubMed, Scopus, and Web of Science which, after duplicate removal, reduced to 1170 records. To this, we added 3 additional records which were found through citation searches. 101 records were subjected to full-text screening. We identified 15 studies that matched our selection criteria (Fig. 1), among them 11 prospective cohort studies and 4 cross-sectional studies, with participants drawn from the general population. The studies varied in size from 97 to 3542 subjects. Tables 1 and 2 show relevant characteristics of the studies included in our review. In the Tables, we separated studies that related IQ measures to pre- and peri-natal cadmium exposures (Table 1) from those that related cognitive development to contemporaneous exposures in childhood (Table 2).

**Table 1:** Characteristics and summary findings of studies that investigated pre- and perinatal cadmium exposures on cognitive development

References	Child age	Sample size	Location	Study design	Confounders accounted for	Exposure measure	Cadmium concentration	Outcome Assessment/IQ test	Observed Effect	Study Evaluation
[18]	5 years	1305	Bangladesh	Cohort	Age, tester, sex, birth order, birth weight, home environment (HOME score), maternal BMI (early pregnancy), maternal IQ, socio-economic status, arsenic, and lead	Maternal urinary Cd	Median (5^th^ and 95^th^ percentile): 0.63 (0.18-2.0) μg/L (specific gravity-adjusted)	WPPSI FSIQ	A doubling of maternal U-Cd inversely associated with FSIQ at 5 years of age.	M/H
[16]	6 months	718	South Korea	Cohort	Gender, birth weight, maternal age and delivery, height, education, family income, breast feeding	Maternal blood Cd	Geometric mean: 1.4 ± 1.7 μg/L in early pregnancy, 1.5 ± 1.4 μg/L in late pregnancy	BSID-II mental development index	Effect modification between Pb and Cd during late pregnancy	M/H
[10]	4 years	385	Spain	Cohort	child's age, quality of neuropsychological test, child's sex, maternal perceptive-performance IQ at child's aged 14 months, maternal verbal IQ at child's aged 4 years, maternal social class, country of birth, mental health, age, child's mood changes and neuropsychological disorder	Maternal urinary Cd twice during pregnancy (1st and 3rd trimester)	Median: 0.55 ng/mL (1st trimester) and 0.53 ng/mL (3rd trimester)	MSCA (McCarthy Scales of Children's Abilities)	No statistically significant association between Cd and MSCA score	H
[14] (follow-up of [16])	5 years	119	South Korea	Cohort	Sex of child, maternal age, educational level of both parents, family income, maternal BMI.	Maternal blood	Mean: 1.49 ± 0.39 μg/L	K-WPPSI (Korean version of the Wechsler preschool and primary Scale of Intelligence, revised edition (WPPSI-R), FSIQ	Association with declining performance IQ, but not cognitive IQ.	L
[23]	5 years	97	China	cohort	Maternal age, BMI, maternal education, family income, passive smoking, Pb	Cd in cord blood	Mean: 0.44 ± 0.43 μg/L	WPPSI	Association between cord blood and VIQ	L
[17]	4 years	575	Greece	Cohort	Examiner, child sex, age at testing (years), and maternal age, parity, marital status, and tobacco smoking (never/ever), urinary lead	Maternal urinary Cd	Mean 0.54± 0.39 μg/L (specific gravity-adjusted)	McCarty Scale of Children's Abilities	Maternal urinary cadmium levels ≥ 0.8 ug/L) inversely associated with children's general cognitive score	M/H
[40]	1 year	149	China	Cross-sectional	Maternal age, IQ, pre-pregnancy BMI, smoking, meat-consumption. Parental education, household income, infant gender, birth weight, gestational age, Pb, Hg, etc.	Maternal blood Cd	Median 4.24 μg/L, range 0.13- 4.55 μg/L	Gesell Development Domains	Lower development quotient in social domain of Gesell Dev. Schedule	H
[11]	4-5 years	302	Spain	Cohort	child gender, psychologist, child age at neuropsychological assessment, social class, maternal smoking during pregnancy, pre-pregnancy BMI	Cd in placenta homogenates	Median 4.1 ng/g	McCarty Scale of Children's Abilities WASI	No association	M/H
[41]	at ages 5 and 8 years	276	USA	Cohort	maternal depression, maternal IQ, sociodemographic factor, exposure to tobacco, lead and ∑PCB	Maternal urinary Cd	Geometric mean 0.17 ± 2.29 μg/g creatinine	Bayley Scale of Infant Development II, the Wechsler Preschool and Primary Scales of Intelligence III and IV	No significant association	M/H
[42]	school age 6-7 years	296	China	Cohort	sex, maternal age at delivery, maternal education level, family annual income, family inhabitation area and passive smoking	cord blood and children's urine	Geometric mean urinary Cd 0.18 ± 2.37 μg/L (specific gravity-adjusted); geometric mean cord blood Cd 0.36 ± 2.01 μg/L	Wechsler Intelligence Scale for Children-Chinese Revised (WISC-CR)	Cord blood Cd and urinary Cd was negatively associated with FSIQ but only in boys; in girls, only urinary Cd was negatively associated with IQ	M/H
[26]	2 years	3542	Japan	cohort	Pre-pregnancy BMI, diabetes, occupation, household income, education smoking, age at delivery, marital status, parity, Pb, Hg	Maternal and cord blood Cd	Maternal blood mean: 0.79 ± 0.39 μg/L; cord blood mean: 0.05 ± 0.02 μg/L	Kyoto Scale of Psychological Development	Maternal blood Cd associated with lower DQ in boys among smoking mothers and those with diabetes	M/H

**Table 2:** Characteristics and summary findings of studies that investigated contemporaneous cadmium exposures on cognitive development in young children

References	Child age	Sample size	Location	Study design	Confounders accounted for	Exposure measure	Cadmium concentration	Outcome Assessment/IQ test	Observed Effect	Study Evaluation
[18]	5 years	1305	Bangladesh	Cohort	Age at testing, tester, sex, birth order, birth weight, HOME, maternal BMI early pregnancy), maternal IQ, socio-economic status, arsenic and lead	Childrens’ urinary Cd	Median (5^th^ and 95^th^ percentile): 0.22 (0.78-0.63) μg/L (specific gravity-adjusted)	WPPSI FSIQ	Concurrent U-Cd in children associated with decreased FSIQ, but weaker than maternal urinary levels	H/M
[33]	6-9 years	261	Spain	Cross sectional	sex, child's age, body mass index (BMI), mother's age, IQ and education, monthly family income, family status, gestational age, vegetables and cereals intake and levels of Mn, As, Pb, Hg in urine	Urinary cadmium	Geometric mean girls: 0.725, boys: 0.769 μg/g creatinine	WISC-IV	Contemporaneous urinary Cd associated with significantly lower scores in FSIQ, stronger among boys	H
[13] (Follow-up of [18])	10 years	1498	Bangladesh	Cohort	Age, sex, birth order, birth weight, HAZ (5 year), HOME, maternal BMI (early pregnancy), maternal IQ, socio economic status, urinary arsenic and lead	Childrens’ urinary Cd	Medians of tertiles: 0.13, 0.24, 0.43 μg/L (specific gravity-adjusted)	WISC-IV	Concurrent urinary cadmium at 10 years of age negatively associated with full scale IQ.	H
[25]	6-12 years	299	Italy	Cross sectional	sex, age, maternal nonverbal intelligence, and cognitive stimulation, home environment, socio economic status	Childrens’ urinary Cd	Median (range) 0.4 ng/mL (0-1.8)	WISC-IV	Contemporaneous urinary Cd was negatively associated with the IQ total score but did not reach statistical significance.	H
[29]	9-11 yr	530	China	Cross sectional	children's age, sex, passive smoking at home, annual family income, parent's education, and occupation	Cord blood and childrens’ urinary Cd	Mean blood Cd: 1.93 μg/L; mean urinary Cd: 1.43 μg/L	CRT-C2 (Combined Raven’s Test in China)	No association with contemporaneous urinary Cd levels, but association may have been obscured by high lead exposure	M

### Study selection and evaluation

Exposure measurements of cadmium that corresponded to prenatal exposures would be ideal for investigating associations with declines in IQ scores as a result of disrupted brain development during gestation. As cadmium has a relatively long half-life of excretion (11 years), maternal urinary cadmium concentrations are a good biomarker of prenatal exposures, along with maternal blood which represents more recent exposures. Four papers related the study outcomes to maternal urinary cadmium concentrations [10, 17, 18, 41], while another four papers analysed cadmium in maternal blood [14, 16, 26, 40]. Five studies used childrens’ urinary cadmium as the exposure marker ([13,25,29, 33, 42]), four analysed cord blood cadmium levels [23, 26, 29, 42] and a single study recorded cadmium in placental or cord blood [11]. One study also examined hair cadmium levels [33].

We rated the exposure assessments in almost all studies as “Good (G)” (Table 3). These studies utilised high standard analytical protocols with a high proportion above the LOD. Liu et al. [23] and Ma et al. [26] did not report the LOD of their methods and we therefore downgraded them to “Adequate (A)” with respect to the exposure domain.

**Table 3:** Confidence rating of 15 eligible studies of associations of cadmium exposure with declines in cognitive ability in children

First author	Exposure	Outcome	Selection	Confounding	Analysis	Overall
[18]	Good	Good	Deficient	Good	Good	**M/H**
[16]	Good	Good	Good	Deficient	Good	**M/H**
[10]	Good	Good	Good	Good	Good	**H**
[33]	Good	Good	Good	Good	Good	**H**
[14]	Good	Good	Deficient	Deficient	Good	**L**
[23]	Adequate	Good	Deficient	Deficient	Good	**L**
[40]	Good	Good	Deficient	Good	Good	**M/H**
[17]	Good	Good	Good	Deficient	Good	**M/H**
[11]	Good	Good	Adequate	Deficient	Good	**M**
[13]	Good	Good	Good	Good	Good	**H**
[29]	Good	Good	Good	Deficient	Good	**M/H**
[25]	Good	Good	Good	Good	Good	**H**
[41]	Good	Good	Deficient	Good	Good	**M/H**
[42]	Good	Good	Good	Deficient	Good	**M/H**
[26]	Adequate	Good	Good	Deficient	Good	**M**

We assessed the outcome measurements in all studies as "Good (G)". The methods employed by most researchers evaluated cognition in children in terms of IQ scores by using versions of the Wechsler intelligence scale (WISC- III and IV, ASI, WISC-CR, WPPSI, K-WPPSI), Gesell, Bayley Scale of Infant Development (BSD, 3rd edition), the Korean version of BSD-II, Mc Carty Scale of Children's Abilities or the CRT-C2 (combined Ravens test in China). The tests were conducted by trained personnel in conducive environments.

In the participant selection evaluation domain, we assigned the rating of “Good (G)” to studies that chose subjects from the general population, with no apparent selection effects and high participation rates and, in prospective cohort studies, with minimum loss to follow up ([10, 13, 17, 42,25, 16, 29, 33]). One study did not give information on loss to follow-up and was therefore downgraded to "Adequate (A)" [11]. We evaluated studies with either low participation rates or significant loss to follow-up as "Deficient (D)" in the participant selection domain [14, 18, 23, 40, 41].

Key confounders that must be considered for declines in IQ studies include maternal age, maternal IQ scores, mother's educational status, socioeconomic status, household income, home environment (HOME score), smoking or alcohol consumption during pregnancy, high-risk pregnancy or other complications, nutritional deficiencies, and stress. Possible genetic predispositions could also be considered but are not well established as risk factors. Of the eligible studies, seven adjusted for key confounders, and accordingly, we evaluated them as "Good (G)" ([10, 13, 18, 25, 33, 40,41]). However, we rated eight studies as “Deficient (D)” in the confounder domain, mostly because they did not include maternal IQ [11, 14, 16, 17, 23, 26, 29, 42].

Finally, all studies used IQ scores (or equivalent) as continuous variables and expressed results in terms of correlation or regression coefficients or beta values and corresponding p-values at 95% confidence intervals. No study reported results as “significant” without supporting quantitative measures. Consequently, we applied a rating of “Good (G)” to the analysis domain of all studies. (Table 3).

### Overall study confidence ratings

Studies with all assessment parameters assessed as “Good (G)” or only one domain classed as “Adequate (A)” achieved an overall confidence rating of "High (H)". With this decision rule, we assigned a confidence rating of “High (H)” to four studies ([10, 13, 25, 33]). Studies with either missing information on maternal IQ or a rating of “Deficient (D)” in the selection domain due to loss of follow up, but with all other domains rated as "Good (G)", were assigned an overall confidence rating of "Medium to High (M/H)". This applied to seven studies [16–18, 29, 40–42]. With one component assessed as "Deficient (D)" and one as “Adequate (A)”, two studies received an overall confidence rating of “Medium (M)” [11, 26]. Where two aspects were classed as "Deficient (CD)", the overall rating was pegged at "Low (L)” [14, 23].

### Evidence synthesis

Of the 10 studies which observed associations of cadmium exposure with child IQ declines, two achieved a confidence rating of “High (H)” [13, 33], five obtained “Medium to High (M/H)” [16–18, 40, 42], while one study received a rating of “Medium (M)” [26]. The low-confidence studies by Liu et al. [23] and Jeong et al. [14] also observed associations.

Five studies did not find significant associations between cadmium exposure and reduced cognitive ability. Of these, we rated two as “High (H)” confidence ([10] and [25]), and two as “Medium to High (M/H)” [29, 41]. One study was classed as “Medium (M)” confidence [11].

The discrepancies in observing associations are partly explained by differences in cadmium exposures. Forns et al. [10] and Yang et al. [41] reported rather low urinary cadmium levels which may have precluded the detection of any associations with declining IQ scores. There were comparatively high exposures to lead in the study by Pan et al. [29] which also may have confounded any effects of cadmium. Lucchini et al. (2019) [25] observed a trend of declining cognitive abilities with increasing proximity to a cadmium-emitting installation in Italy, but these did not reach statistical significance.

With two “High (H)” confidence, five “Medium to High (M/H)” confidence and one “Medium (M)” confidence study demonstrating associations of cadmium exposures with declines in childrens’ IQ loss, the overall strength of evidence can be evaluated as “Robust (R)”.

### Derivation of a reference dose for IQ loss

We used the high-confidence study by Gustin et al. [13] to estimate cadmium exposures no longer associated with compromised cognitive development. In this study, the authors examined urinary cadmium levels and cognitive abilities and behaviour of 10-year-old children as a follow up study of the mother-child cohort by Kippler et al. [18]. To avoid potential bias due to comparisons with foreign culture norms, Gustin et al. used the WISC-IV raw scores without adjusting different scales to a mean of 100 (U.S. norms). They adjusted for arsenic and lead urinary levels and stratified the population of 10-year-old children according to tertiles of specific gravity-adjusted urinary cadmium concentrations, with referents in the lowest tertile (median: 0.13 μg/L, range 0.016 – 0.19 μg/L), 0.24 μg/L (median, range: 0.19 – 0.31 μg/L) in the second tertile and 0.43 μg/L (median, range: 0.31 – 2.6 μg/L) in the highest tertile. Urinary cadmium levels in 10-year-olds were associated with poorer cognitive abilities, with children in the highest tertile of urinary cadmium levels performing 4.7 scores lower in the Full-Scale IQ compared with children in the lowest tertile. The effect was more pronounced in boys (7.0 scores lower).

Gustin et al. emphasised that the inverse relationship between urinary cadmium and Full-Scale IQ was without an apparent threshold but observed a statistically significant association between urinary cadmium and diminished IQ only in the highest tertile. To estimate a reference dose for declines in cognitive abilities (epidemiological “no-observed-adverse-effect level”), we therefore considered the childrens’ urinary cadmium levels in the second tertile. We reasoned that to avoid experiencing cadmium-related IQ deteriorations, urinary cadmium concentrations in 10-year-olds must not exceed the range of 0.19 – 0.31 μg/L. We used the one-compartment toxicokinetic model developed by Amzal et al. [1] to derive daily intakes that after 10 years of life would produce such urinary cadmium concentrations.

The Amzal model accounts for the half-life of excretion of cadmium, its absorption from the gastro-intestinal tract and transport to the kidney and the age of the children. We first assumed a half-life of excretion of 11.3 years, and a value of 0.005 for constants related to the absorption of cadmium, its transport to the kidney and the ratio of cadmium in urine to its concentration in the kidney cortex (fu x fk) [1]. The model works based on creatine-adjusted urinary cadmium levels, but the data given in Gustin et al. [13] are specific gravity-adjusted. Suwazono et al. [36] showed that creatinine-adjusted and specific gravity-adjusted urinary cadmium are comparable in subjects under 50 years of age. On this basis, we used the specific gravity-adjusted values in Gustin et al. [13].

Based on a half-life of excretion of 11.3 years, we estimated that urinary cadmium levels of 0.19, 0.24 and 0.31 μg/L will be reached after 10 years of exposure to 0.29, 0.37 and 0.48 μg/kg/d, respectively. However, there is considerable inter-individual variation in the half-life of excretion, which in some individuals extends to 20 years and longer [1]. With a half-life of 20 years, correspondingly lower daily intakes of 0.15, 0.19 and 0.25 μg/kg/d will lead to urinary cadmium levels of 0.19, 0.24 and 0.31 μg/L in 10-year-old children, respectively. To account for individuals with long cadmium excretion times, we therefore propose a daily intake of 0.2 μg/kg/d as a cadmium reference dose for assessing cognitive declines in a mixture risk assessment.

Our reference dose estimate is 1.8-fold lower than the current European Food Safety Agency (EFSA) health-based guidance value of 0.36 μg/kg body weight/day which was derived based on kidney toxicity [7].

### Comparison with estimated daily intakes in Europe

In 2012, the European Food Safety Authority [8] provided estimates of the lifetime cadmium dietary exposure of the European population. According to this report, the middle bound overall weekly average intake was estimated as 2.04 μg/kg body weight with a 95^th^ percentile at 3.66 μg/kg body weight. These figures are equivalent to 0.29 and 0.52 μg/kg body weight/day, respectively, and are 1.45 to 2.6 times higher than our proposed reference dose for declines in IQ of 0.2 μg/ kg body weight/day.

### Discussion

There has been significant progress in elucidating associations between cadmium and deteriorations of cognitive ability since Rodríguez-Barranco et al. [32] evaluated the evidence as inconclusive. Already by 2018, a considerable number of studies had appeared that demonstrated associations between cadmium and cognitive declines in children, predominantly because of prenatal exposures. This body of data led Liu et al. [24] to conclude that there is convincing evidence of harmful effects on the cognitive abilities of offspring. Since then, additional studies, not reviewed by Liu et al. [24], have strengthened the evidence even further [26, 42]. Although we utilised different criteria in our risk-of-bias evaluation than those employed by Liu et al. [24] (who made use of the Newcastle-Ottawa Scale), we arrived at similar confidence ratings. In summary, the evidence linking prenatal, perinatal, and early childhood exposures to cadmium with declines in cognitive ability can be evaluated as robust. Already in 2012, scientists have pointed to cadmium as the “new lead”, due to widespread exposure in children [33]. The data that accumulated since then substantiate this view.

This assessment is not shared by Lamkarkach et al. [21]. In their effort of deriving human biomonitoring values for cadmium as part of the European Joint Programme on Human Biomonitoring (HBM4EU), they evaluated data on the association of cadmium with neurotoxic effects as limited and concluded that neurological endpoints cannot be regarded as critical effects of cadmium. However, Lamkarkach et al. [21] did not take account of the systematic review by Liu et al. [24] and based their assessment only on the papers by Kippler et al. [18], Rodríguez-Barranco et al. [33], Ciesielski et al. [5], Cao et al. [3] and Gustin et al. [13], without noting the remainder of the studies reviewed by us and by Liu et al. [24].

The goal of most studies considered here was in learning about a causal role of cadmium for declines in cognitive ability. Such research uses regression models for causal inferences with the intention of controlling the possible confounding role of other factors such as lead or mercury exposures, the home environment, etc. Although the coefficients used in these regression models allow statements such as “a doubling of cadmium concentration in urine leads to an x-fold decrease in IQ scores”, this information does not always permit estimations of “epidemiological” NOAELs because the data needed for a positioning of the regression models along the dose axis are not necessarily accessible from the published papers. This constrained our efforts of estimating a reference dose for cognitive declines, as only a few studies contained the required data.

With these provisos, the “High (H)”-confidence study by Gustin et al. [13] seemed most appropriate for our purpose. Effects were seen in 10-year-old children, suggesting that they were not compensated through the home environment or schooling, and therefore more likely to be permanent. Our approximation of a daily cadmium intake of 0.2 μg/kg/d as no longer associated with cognitive declines is based on the statistical significance of associations observed by Gustin et al. [13] in tertiles of urinary cadmium levels. Comparison with the data from Kippler et al. [17] of a mother-child cohort in Crete is instructive. In this study, maternal urinary cadmium levels below 0.8 μg/L (specific gravity-adjusted) were no longer associated with the cognitive ability of 4-year-old children. Exposure to 0.29 μg/kg/d over 30 years (the median maternal age in this cohort) can be expected to lead to such urinary cadmium concentrations, assuming a half-life of excretion of 20 years (Amzal model). Both this, and the estimate derived from Gustin for children are below the cadmium HBGV of 0.36 μg/kg/d [7]. It would therefore appear that the current HBGV is insufficiently protective against neurodevelopmental effects of cadmium and will be unsuitable for use in a mixture risk assessment for cognitive declines. We therefore propose the application of a value of 0.2 μg/kg/d for this purpose.

However, our estimate of 0.2 μg/kg/d must be treated with caution as Gustin et al. [13] stressed they saw no evidence of a dose threshold. We therefore emphasise that our value does not meet the standards required for derivation of a new HBGV, nor is this our intention here. Our interest is in arriving at a reasonable estimate for use in a mixture risk assessment, without the normative character associated with a HBGV. A revision of the HBGV will require a thorough quantitative evaluation of available data including benchmark dose modelling, all beyond our resources due to lack of access to raw data. However, we strongly recommend that the relevant authorities and agencies consider the case of cadmium developmental neurotoxicity and its implications for a revised HBGV.

## Conclusion

In conclusion, prenatal and early childhood exposures to cadmium are associated with deteriorations in childrens’ cognitive functions. Although the observed effect magnitudes may be immaterial at an individual level, the impact on populations is likely to be substantial. Based on the available evidence, we propose a reference dose of 0.2 μg/kg body weight/d for cadmium in mixture risk assessments of declines in cognitive ability.

## Data Availability

Not Applicable

## Abbreviations

AAS: Atomic Absorption Spectroscopy
ADHD: Attention Deficit Hyperactivity Disorder
BMI: Body Mass Index
BSD: Bayley Scale of Development
COSTER: Conduct of Systematic Reviews in Toxicology and Environmental Health Research
CRT: Combined Ravens Test
EFSA: European Food Safety Authority
FSIQ: Full Scale Intelligence Quotient
HBGV: Health-based guidance value
HI: Hazard Index
HOME: Home Observation for Measurement of the Environment
ICPMS: Inductively Coupled Plasma Mass Spectrometry
IQ: Intelligence Quotient
LOD: Limit of Detection
μg/kg: microgram per kilogram
MSCA: McCarthy Scales of Childrens’ Abilites
NOAEL: No Observed Adverse Effects Level
PECO: Population Exposure Comparison Outcome/Observation
PCB: Polychlorinated Biphenyls
QA: Quality Assurance
RoB: Risk of Bias
t1/2: half life
WISC: Weschler intelligence scale
WPPSI: Weschler Preschool and Primary Scale of Intelligence
